# Supplementary material for: Exposure to trauma in pregnant women and its association with previous perinatal complications, IPV and antenatal service satisfaction in rural Ethiopia: a cross-sectional facility-based study
Source: PLoS One. 2025 Sep 5;20(9):e0319362. doi: 10.1371/journal.pone.0319362 (PMC12413002; doi:10.1371/journal.pone.0319362)
Supplement: S1 Table — (DOCX) [file pone.0319362.s001.docx]

S1 Table. Associations between PCL-5 total score mental health disorders, IPV obstetric complications and ANC satisfaction.

|  | Unadjusted mixed-effects linear regression  Regression coefficient (RC) 95% confidence interval (CI) | Adjusted model for parity, age, education and area of residence  Adjusted RC (95%CI) |
| --- | --- | --- |
| Probable depression | 14.43 (12.04 – 16.83) *** | 14.41 (11.94-16.85) *** |
| Moderate to severe Anxiety symptoms | 29.96 (26.80- 33.11) *** | 29.87 (26.73- 33.00) *** |
| Probable IPV | 7.86 (5.32- 10.40) *** | 7.79 (5.20-10.39) *** |
| Obstetric complications | 1.30 (-0.33 – 2.92) | 1.23 (-0.30 – 2.77) |
| Total satisfaction score with ANC | -0.18 (-0.22 – (-0.14)) *** | -0.18 (-0.22- (-0.13)) *** |

*** p<0.001
