# Supplementary material for: Exposure to trauma in pregnant women and its association with previous perinatal complications, IPV and antenatal service satisfaction in rural Ethiopia: a cross-sectional facility-based study
Source: PLoS One. 2025 Sep 5;20(9):e0319362. doi: 10.1371/journal.pone.0319362 (PMC12413002; doi:10.1371/journal.pone.0319362)
Supplement: Attachment — Submitted filename: PLOS Review.docx [file pone.0319362.s003.docx]

The topic is of high public health significance, particularly in LMICs where perinatal mental health is often neglected despite its profound intergenerational effects. The study highlights a gap in literature on perinatal PTSD in African settings, moving beyond the common focus on depression and anxiety. However, there are some suggestions which can strength the manuscript.

In my view, cross sectional design limits the ability to draw causal inferences between trauma exposure, PTSD, and ANC satisfaction. Longitudinal data would be better suited to assess the trajectory and impacts of PTSD over time. Potential reporting bias were not properly addressed s sensitive topics such as IPV and mental health symptoms were self-reported and may be underreported due to stigma or fear, especially in patriarchal rural settings. While the PCL-5 was used, the absence of clinical diagnostic interviews may have led to misclassification. The cut-off of 31, though standard, may need local validation.

The satisfaction scale was originally designed for mental health services. Its adaptation to ANC settings is not fully described—how well it captured maternity-specific concerns is unclear.

The categorization of IPV based on just three items may miss subtler or more severe forms of abuse. The scale might underestimate IPV prevalence. There is no mention of how missing data were handled, which could influence the validity of the results. The study presents **valuable, methodologically sound findings** that advance understanding of trauma and PTSD among pregnant women in a resource-limited context.

There is no mention of theoretical models (e.g., trauma-informed care frameworks, health-seeking behavior models) that could strengthen the interpretation of complex findings. This weakens the scholarly rigor of the discussion even it should be part of methodology.

The discussion briefly notes that PTSD prevalence was low, but it misses a deeper exploration of whether this reflects methodological limitations (e.g., time window of trauma, cultural variation in symptom reporting, or cutoff scores). The conclusion would benefit from a more nuanced interpretation of this discrepancy.

The observation that women with probable PTSD scored some aspects of ANC more positively is raised but not critically interrogated. Is this a reflection of lowered expectations, social desirability bias, or misinterpretation of the scale? These ambiguities weaken the interpretation. The authors rightly emphasize person-centred maternity care, but the discussion lacks depth about how this can be implemented effectively in the Ethiopian context. There is also limited integration of study findings to support this advocacy—what specific elements of care were deficient?

Finally, conclusion appropriately emphasizes the need for better recognition of trauma and integration of psychosocial care into ANC, but falls short in providing **actionable insight or priorities** for intervention or future research.
