# Supplementary material for: Exposure to trauma in pregnant women and its association with previous perinatal complications, IPV and antenatal service satisfaction in rural Ethiopia: a cross-sectional facility-based study
Source: PLoS One. 2025 Sep 5;20(9):e0319362. doi: 10.1371/journal.pone.0319362 (PMC12413002; doi:10.1371/journal.pone.0319362)
Supplement: Attachment — Submitted filename: Response to Reviewers.docx [file pone.0319362.s005.docx]

Muhammad Haroon Stanikzai
Academic Editor
PLOS ONE

9^th^ June 2025

Dear Dr Stanikzai,

Re:	PONE-D-25-05649

**Exposure to trauma in pregnant women and its association with previous perinatal complications, IPV and antenatal service satisfaction in rural Ethiopia: a cross-sectional facility-based study**

We thank reviewers for their thoughtful comments and opportunity to address their points and suggestions to improve the current manuscript. We include point by point responses to reviewer comments below.

In response to your Editor comments:

1. We have revised the formatting as per PLOS guidelines.
2. We have completed and uploaded the
3. We confirm that the funders had no role in study design, data collection and analysis, decision to publish, or preparation of the manuscript.
4. With respect to data sharing, we stated in the original submission that there would be no restriction to accessing data underlying analyses presented in the paper. All survey database files are available from Open Science Framework database (<https://osf.io/7s6wv/>).
5. We have corrected the authorship list.
6. We have amended the Figure to be a Supplementary File.
7. We have reviewed the reference list.

Thank you for this opportunity to revise our manuscript. Please do not hesitate to get in touch if any further clarifications are required.

Yours sincerely

Professor Charlotte Hanlon

**Response to reviewers**

1. The topic is of high public health significance, particularly in LMICs where perinatal mental health is often neglected despite its profound intergenerational effects. The study highlights a gap in literature on perinatal PTSD in African settings, moving beyond the common focus on depression and anxiety. However, there are some suggestions which can strength the manuscript.

*RESPONSE: Thank you for your comments to improve our manuscript.*

2. In my view, cross sectional design limits the ability to draw causal inferences between trauma exposure, PTSD, and ANC satisfaction. Longitudinal data would be better suited to assess the trajectory and impacts of PTSD over time.

*RESPONSE: We recognize the limitation of our cross-sectional analysis. In the section “Strengths and Limitations” we have now added the following sentence: “Due to the cross-sectional design of our study, we are also unable to draw casual inferences; studies with a longitudinal design would be helpful to further identify the trajectory of trauma symptoms and their impact on psychological wellbeing and ANC satisfaction over time in this setting.” (page 35).*

3. Potential reporting bias were not properly addressed s sensitive topics such as IPV and mental health symptoms were self-reported and may be underreported due to stigma or fear, especially in patriarchal rural settings. While the PCL-5 was used, the absence of clinical diagnostic interviews may have led to misclassification. The cut-off of 31, though standard, may need local validation.

*RESPONSE: We recognize the sensitivity of the topic and challenges with accurate measurement and have amended the “Strengths and Limitations” section on page 34 as follows: “Rural populations in Ethiopia face multiple barriers to care including low awareness of, and high stigma, related to mental health symptoms(46,47). Previous studies have shown that females and those with lower level of education have higher internalised self-stigma regarding mental illness in the country(48). Therefore, fear and stigma of sensitive topics such as IPV, trauma and mental health symptoms may have contributed to significant underreporting in our study. This is the first study in Ethiopia and one of few from LMICs, to investigate the relationships between trauma symptoms, IPV, obstetric complications and ANC satisfaction using contextually validated (for satisfaction) and adapted (trauma, IPV) measures in a large sample. Limitations include potential under detection of traumatic events, PTSD and IPV. There is a need for further studies to examine how trauma symptoms may manifest differently in this setting, the validity of cut-off points used by our measuring tools and to develop targeted interventions to elicit sensitive disclosure. “*

*We acknowledge the limitation that cut-off of 31 in the PCL-5 was not validated in this setting. In recognition of that, we used the term “probable PTSD” throughout the paper. In addition, we have now conducted further analyses using the PCL-5 total score as continuous measure which corroborate the direction of the associations found using the cut off score of 31 (supplementary table 1). To reflect the extra analyses, we added “We also conducted linear mixed-effects regression tests of association between PCL-5 total scores and co-morbid depression, anxiety, probable IPV and total ANC satisfaction score*.” *in Analysis Plan on page 10 and reported results on pages 17 and 18.*

4. The satisfaction scale was originally designed for mental health services. Its adaptation to ANC settings is not fully described—how well it captured maternity-specific concerns is unclear.

*RESPONSE: We have now included the following statement in the “Strengths and Limitations” section on page 34: “The satisfaction scale used was initially developed to capture satisfaction with mental health services and although it includes several of the relevant concepts for person-center care including dignity, respect and collaboration, it may be missing other important aspects of maternity-specific care.”*

5. The categorization of IPV based on just three items may miss subtler or more severe forms of abuse. The scale might underestimate IPV prevalence.

*RESPONSE*: *We thank the reviewer for raising this point. However, work by Zink et al showed that less direct ways of indicating IPV may be more accurate, and that the three-question combination including the domains of argument, safety, and manner of treating the responder had the best results (Zink T, Levin L, Putnam F, Beckstrom A. Accuracy of Five Domestic Violence Screening Questions With Nongraphic Language. Clinical Pediatrics. 2007;46(2):127-134. doi:*[*10.1177/0009922806290029*](https://doi.org/10.1177/0009922806290029)*).*

6. There is no mention of how missing data were handled, which could influence the validity of the results.

*RESPONSE: We thank reviewer for raising this point. We now added the sentence “Complete case analyses were performed.” to the analysis plan section on page 10.*

7. The study presents **valuable, methodologically sound findings** that advance understanding of trauma and PTSD among pregnant women in a resource-limited context.

*RESPONSE: We thank the reviewer for the positive feedback and opportunity to address their comments.*

8. There is no mention of theoretical models (e.g., trauma-informed care frameworks, health-seeking behavior models) that could strengthen the interpretation of complex findings. This weakens the scholarly rigor of the discussion even it should be part of methodology.

*RESPONSE: In order to strengthen our interpretation and highlight commonalities between the endorsed model of person-centered care and trauma informed care we added the following sentence:* *“In higher income settings, trauma-informed approaches have been increasingly cited in policy and adopted in practice as a means for reducing the negative impact of trauma experiences and supporting mental and physical health outcomes and prevent re-traumatisation (48). Whereas several of the principles of person-centered care overlap with principles of trauma-informed care (such as trustworthiness, safety, collaboration and choice), further studies are required to understand how local policies, protocols and processes can be responsive to the needs of individuals served and how they can be empowered in their interactions with the health system.“ (pages 34 and 35).*

9. The discussion briefly notes that PTSD prevalence was low, but it misses a deeper exploration of whether this reflects methodological limitations (e.g., time window of trauma, cultural variation in symptom reporting, or cutoff scores). The conclusion would benefit from a more nuanced interpretation of this discrepancy.

*RESPONSE: In response to these important comments raised by the author we have modified the “Strengths and Limitations” section of our manuscript on page 34 as follows: “Rural populations in Ethiopia face multiple barriers to care including low awareness of, and high stigma, related to mental health symptoms(46,47). Previous studies have shown that females and those with lower level of education have higher internalised self-stigma regarding mental illness in the country(48). Therefore, fear and stigma of sensitive topics such as IPV, trauma and mental health symptoms may have contributed to significant underreporting in our study. This is the first study in Ethiopia and one of few from LMICs, to investigate the relationships between trauma symptoms, IPV, obstetric complications and ANC satisfaction using contextually validated (for satisfaction) and adapted (trauma, IPV) measures in a large sample. Limitations include potential under detection of traumatic events, PTSD and IPV. There is a need for further studies to examine how trauma symptoms may manifest differently in this setting, the validity of cut-off points used by our measuring tools and to develop targeted interventions to elicit sensitive disclosure. “ and “Due to the cross-sectional design of our study, we are also unable to draw casual inferences; studies with a longitudinal design would be helpful to further identify the trajectory of trauma symptoms and their impact on psychological wellbeing and ANC satisfaction over time in this setting.” on page 35. We also acknowledge the limitation that cut-off of 31 in the PCL-5 was not validated in this setting. In recognition of that, we used the term “probable PTSD” throughout the paper. In addition, we have now conducted further analyses using the PCL-5 total score as continuous measure which corroborate the direction of the associations found using the cut off score of 31 (supplementary table 1). To reflect the extra analyses, we added “We also conducted linear mixed-effects regression tests of association between PCL-5 total scores and co-morbid depression, anxiety, probable IPV and total ANC satisfaction score*.” *in Analysis Plan on page 10 and reported results on pages 17 and 18.*

10. The observation that women with probable PTSD scored some aspects of ANC more positively is raised but not critically interrogated. Is this a reflection of lowered expectations, social desirability bias, or misinterpretation of the scale? These ambiguities weaken the interpretation.

*RESPONSE: The reviewer made an important point regarding the need to further interrogate these findings. We amended the paragraph in “Discussion” in page 32 as following to provide a more nuanced interpretation:* “*Women with antenatal PTSD showed a lower overall satisfaction with ANC compared to women without antenatal PTSD but paradoxically scored certain aspects of care higher than women without PTSD. Indeed, they endorsed more items regarding respect for their privacy and confidentiality. This finding should be interpreted with caution due to potential for response bias if women believe their answers may influence further care. In addition, previous studies in lower income countries have shown that lower expectations can lead to higher levels of satisfaction with care(38). A study from Ethiopia also reported higher satisfaction with ANC care to be associated with lower levels of education(39) and women in our sample with antenatal PTSD were more likely to have lower levels of education than those without, highlighting the complex interplay between education, expectations and potential social desirability bias (the tendency to underreport socially undesirable attitudes and behaviors and to over report more desirable attributes). “*

11. The authors rightly emphasize person-centred maternity care, but the discussion lacks depth about how this can be implemented effectively in the Ethiopian context. There is also limited integration of study findings to support this advocacy—what specific elements of care were deficient? Finally, conclusion appropriately emphasizes the need for better recognition of trauma and integration of psychosocial care into ANC, but falls short in providing **actionable insight or *priorities*** *for intervention or future research.*

*RESPONSE: Thank you for highlighting the need to identify priorities for intervention. We have added the following paragraph to “Strengths and Limitations on page 35: ” Future research should explore women’s, maternal health care workers’ and other relevant stakeholders’, including intimate partners’, perspectives on high quality ANC and person-centred care and specifically how services can be adapted to ensure the necessary space is provided to facilitate sensitive disclosure of trauma, validate feelings and concerns of staff and patients, support people to make decisions and take action and involve family members in a constructive and culturally-acceptable manner. Recently, there has been close working between the Ethiopian Federal Ministry of Health and the ASSET research team to integrate the Practical Approach to Care Kit (PACK) programme, which comprises the provision of carefully designed comprehensive and integrated clinical decision support into primary health care (50)and develop training on person-centered maternal care(51). In addition, there is ongoing research to support the development of brief psychological interventions for perinatal women with exposure to IPV that can be feasibly delivered by primary care workers(52,53).”*

We hope the current revision addresses all the raised points by reviewers and we remain committed to improve the manuscript to the highest standard by addressing the constructive feedback given.
