# Supplementary material for: Exposure to trauma in pregnant women and its association with previous perinatal complications, IPV and antenatal service satisfaction in rural Ethiopia: a cross-sectional facility-based study
Source: PLoS One. 2025 Sep 5;20(9):e0319362. doi: 10.1371/journal.pone.0319362 (PMC12413002; doi:10.1371/journal.pone.0319362)
Supplement: Attachment — Submitted filename: Response to Reviewers_2025_07_01.docx [file pone.0319362.s006.docx]

Muhammad Haroon Stanikzai
Academic Editor
PLOS ONE

1^st^ July 2025

Dear Dr Stanikzai,

Re:	PONE-D-25-05649

**Exposure to trauma in pregnant women and its association with previous perinatal complications, IPV and antenatal service satisfaction in rural Ethiopia: a cross-sectional facility-based study**

Thank you for the opportunity to further review and improve our manuscript. We addressed the raised points as below:

*Journal Requirements:
Please review your reference list to ensure that it is complete and correct. If you have cited papers that have been retracted, please include the rationale for doing so in the manuscript text, or remove these references and replace them with relevant current references. Any changes to the reference list should be mentioned in the rebuttal letter that accompanies your revised manuscript. If you need to cite a retracted article, indicate the article’s retracted status in the References list and also include a citation and full reference for the retraction notice.*

**RESPONSE:** We have now reviewed the full reference list to ensure references are complete and correct. The following changes have been implemented:

- Reference 14 which was published as pre-print at time of submission of our manuscript but has since been accepted and published has been added as full reference as follows:

14. Eshetu T, Fekadu E, Abdella A, Mulushoa A, Medhin G, Belina M, et al. Towards person-centred maternal and newborn care in Ethiopia: a mixed method study of satisfaction and experiences of care. BMC Pregnancy and Childbirth. 2025; 25(1):1–13. doi: 10.1186/s12884-024-07116-4.

- Reference 21 was updated to a more relevant paper of the validation of GAD-7 as measuring tool for anxiety symptoms.

21. Spitzer RL, Kroenke K, Williams JBW, Löwe B. A Brief Measure for Assessing Generalized Anxiety Disorder: The GAD-7. Arch Intern Med. 2006; 166(10):1092–7. doi:10.1001/archinte.166.10.1092

- Similarly. reference 24 was amended to a more relevant paper describing the use of our intimate partner violence measuring tool in the local setting:

24. Bitew T, Keynejad R, Honikman S, Sorsdahl K, Myers B, Fekadu A, et al. Stakeholder perspectives on antenatal depression and the potential for psychological intervention in rural Ethiopia: A qualitative study. BMC Pregnancy Childbirth. 2020; 20(1):1–11. doi:10.1186/s12884-020-03069-6.

*Additional Editor Comments:
- Please look at previous publications at PLOS ONE and use in-text citations as per journal style.*

**RESPONSE:** We have added in-text citations as per journal style including use of square brackets for references.

*- Please use capital letter for the word of heading and subheadings Please review the whole manuscript and table headings).*

**RESPONSE:** We have now reviewed the whole manuscript and implemented 3 levels of Headings with font size and bold formatting as specified. We have also added Fig 1 caption in the manuscript as well as specified supplementary information at end of manuscript.

*- Line 214: Please make it statistical analysis.*

**RESPONSE:** Changed as requested.

*- The manuscript would be benefit from careful reading by authors (particularly capitalization and punctuation).*

**RESPONSE:** Reviewed as requested. Minor changes were tracked in manuscript.

We hope this version meets the required criteria for publication in your journal.

Kind regards,

Professor Charlotte Hanlon
